# Rationale and design of ‘discontinuing statins in multimorbid older adults without cardiovascular disease (STREAM)’: study protocol of a randomised non-inferiority clinical trial

**DOI:** 10.1136/bmjopen-2024-093833

**Published:** 2025-05-23

**Authors:** Philipp Stefan Aebi, Luise Adam, Moa Haller, Julia Bianca Bardoczi, Baris Gencer, Fabrice Bonnet, Jürg-Hans Beer, Sebastian Carballo, Mirjam Christ-Crain, Martin Feller, Luca Gabutti, Alan G Haynes, Elisavet Moutzouri, Patricia Orializ Chocano-Bedoya, Stefano Bassetti, Robert Escher, Martin Egger, Rosalinde K E Poortvliet, Philipp Schuetz, Sven Trelle, Maria M Wertli, Dina Zekry, Marie Méan, Drahomir Aujesky, Douglas Bauer, Manuel R Blum, Nicolas Rodondi

**Affiliations:** 1 Department of General Internal Medicine, Inselspital, Bern University Hospital, University of Bern, Bern, Switzerland; 2 Institute of Primary Health Care (BIHAM), University of Bern, Bern, Switzerland; 3 Department of Internal Medicine, Angiology, Kantonsspital Schaffhausen, Schaffhausen, Switzerland; 4 Department of Cardiology, University of Lausanne, Lausanne, Switzerland; 5 Service de Médecine Interne et Maladies Infectieuses, Hôpital Saint-André, CHU de Bordeaux, and INSERM U1219, Bordeaux Population Health, Université de Bordeaux, Bordeaux, France; 6 Department of Internal Medicine, Cantonal Hospital of Baden, Baden, Switzerland; 7 University of Zürich, Zürich, Switzerland; 8 Division of General Internal Medicine, Hôpitaux Universitaires Genève, Geneva, Switzerland; 9 Department of Endocrinology, Diabetology and Metabolism, University Hospital Basel, Basel, Switzerland; 10 Department of Internal Medicine, Repubblica e Cantone Ticino Ente Ospedaliero Cantonale, Bellinzona, Switzerland; 11 CTU Bern, Department of Clinical Research, University of Bern, Bern, Switzerland; 12 Division of Internal Medicine, University Hospital Basel, Basel, Switzerland; 13 Department of Internal Medicine, Spital Emmental, Burgdorf, Switzerland; 14 Clinic of Internal Medicine, Spital Emmental, Langnau, Switzerland; 15 Department of Public Health and Primary Care, Leiden University Medical Center, Leiden, The Netherlands; 16 LUMC Center for Medicine for Older people, Leiden University Medical Center, Leiden, The Netherlands; 17 Division of Internal Medicine, Kantonsspital Aarau, Aarau, Switzerland; 18 Division of Internal Medicine for the Aged, Hôpitaux Universitaires Genève, University of Geneva, Geneva, Switzerland; 19 Division of Internal Medicine, Department of Medicine, Lausanne University Hospital, Lausanne, Switzerland; 20 Department of Medicine, University of California, San Francisco, San Francisco, California, USA

**Keywords:** Patients, Lipid disorders, Primary Prevention, Cardiovascular Disease, GERIATRIC MEDICINE, Primary Health Care

## Abstract

**Introduction:**

Statins are among the most widely used drugs. While they are effective for primary and secondary prevention of cardiovascular (CV) disease in middle-aged subjects, their benefits for prevention in older adults (aged ≥70 years) without CV disease are uncertain, particularly for those with multimorbidity. Statin side effects and drug interactions are common in older patients and may negatively impact quality of life. To date, the only randomised controlled trial (RCT) investigating statin discontinuation in older adults has demonstrated no difference in survival but did note a small improvement in quality of life for those who discontinued statins. However, this trial exclusively enrolled patients with a life expectancy <1 year. Therefore, the present RCT aims to assess the safety and potential benefits of statin discontinuation in primary prevention for the ever-growing population of multimorbid older adults.

**Methods and analysis:**

This study is a multicentre, randomised, non-inferiority trial conducted in both inpatient and outpatient settings in Switzerland, France and the Netherlands, targeting patients using statins for primary prevention. 1800 participants are randomly assigned 1:1 to either discontinue (intervention arm) or continue (control arm) statin therapy. The primary objective is to compare the primary composite endpoint of major CV events (non-fatal myocardial infarction or non-fatal ischaemic stroke) and all-cause death between the control and intervention groups over a follow-up duration of up to 48 months. We hypothesise that discontinuing statins does not result in shorter event-free survival, with a non-inferiority margin set at 5.2 weeks over a 2-year observation period. Secondary objectives are to compare patient-centred outcomes (health-related quality of life, muscle pain symptoms, falls and sarcopenia) and all-cause death, non-CV death, major CV events and coronary and peripheral artery revascularisation. The study is open-labelled, with blinded outcome adjudication of the primary endpoints.

**Ethics and dissemination:**

The trial protocol has received approval from the local ethics committees in Switzerland, France and the Netherlands. Results will be published in a peer-reviewed journal.

**Trial registration number:**

Clinicaltrials.gov: NCT05178420; BASEC (Swiss Ethics Commission): 2021-01513; FOPH (Swiss national portal): SNCTP000005172; Netherlands Trial Register: NL83907.058.23; France Trial Register: 22.04747.000158– IDRCB 2022-A02481-42.

**STRENGTHS AND LIMITATIONS OF THIS STUDY**

- This study is the largest ongoing trial to assess the safety and potential benefit of statin discontinuation in multimorbid older adults, who are often excluded from major randomised controlled trials.
- In addition to cardiovascular endpoints, this trial assesses patient-centred outcomes important for older adults, such as quality of life, muscle pain, sarcopenia and falls as secondary endpoints.
- While outcome adjudication and ascertainment is blinded, the lack of participant and physician blinding may introduce bias in treatment adherence and subjective outcome assessments.
- We engaged patients in safety monitoring, recruitment strategies and dissemination efforts, which increases the study’s relevance and applicability in real-world settings.

## Introduction

### Background

 Statins are among the most widely used drugs.^1^ While they have been found to be effective for primary and secondary prevention of cardiovascular (CV) disease (CVD) in middle-aged subjects,^2^,^4^ their benefits for prevention in older adults without CVD remain uncertain, particularly for those over 70 years,^3 4^ and in those with several chronic diagnoses (multimorbidity) who are often excluded from clinical trials.^5^,^7^ First, among those aged ≥70 years, no consistent statin benefit for primary prevention was shown on all-cause mortality and CVD in randomised controlled trials (RCTs).^3 4 8^ Second, the majority of older adults suffer from multimorbidity (commonly defined as ≥2 chronic conditions),^9 10^ and most large RCTs on lipid-lowering therapies did not include multimorbid older adults.^6^ Moreover, individuals aged ≥70 years are under-represented in most RCTs, including those assessing statin benefits for prevention.^68 11^,^16^ Additionally, statin side effects and drug interactions are more likely to occur in older adults and patients with multiple medications and may negatively impact health-related quality of life (QOL).^17^,^21^ The proportion of older patients developing muscle pain under statins has been shown to be 5–20% in observational studies.^17 22 23^

While data supporting statin prescription for primary prevention in older multimorbid adults are limited, in practice, statins are often discontinued in multimorbid older adults without CVD due to side effects.^24^ Recent guidelines on this topic are conflicting. The American Heart Association/American College of Cardiology (AHA/ACC) Cholesterol Guidelines suggest that discontinuing statins may be reasonable when ‘functional decline, multimorbidity, frailty or reduced life-expectancy limits the potential benefits of statins’.^25^ Conversely, other guidelines consider statins in older adults with high CV risk while accounting for factors like frailty and patient preferences. They recognised that there are uncertainties about the benefits and harms of statins in those aged 75 years and older with comorbidities and cognitive impairment.^26^,^28^ Those are, however, expert opinions, not based on data from RCTs on statin discontinuation.

To this day, no RCT examining the benefits of statins in primary prevention has exclusively recruited older (≥70 years) multimorbid participants^6 29^; only a single RCT including participants with a short life expectancy (<1 year) found no difference in CV events and mortality between the groups, but found a slightly better health-related QOL among those who discontinued statins (mean McGill QOL score, 7.11 vs 6.85; p=0.04).^18^ Conversely, in a retrospective cohort study, older adults who discontinued statins for primary prevention were more often admitted to the hospital for a CV event, but interpretation of this data does not allow a causal conclusion, given the design and the lack of measurement of several CVD confounding factors and risk of confounding by indication, given that reasons for statin discontinuation were not reported,^30^ as illustrated by another retrospective cohort study with discontinuation. associated with a higher rate of CV events, but also larger risk of hip fractures and non-CV mortality.^31^ A recent literature review highlighted the importance of further RCTs.^32^

Therefore, the primary aim of this trial is to evaluate the safety of discontinuing versus continuing statin therapy in older adults with multimorbidity but no clinical CV disease.

## Methods and analysis

### Trial design

**Figure 1:**
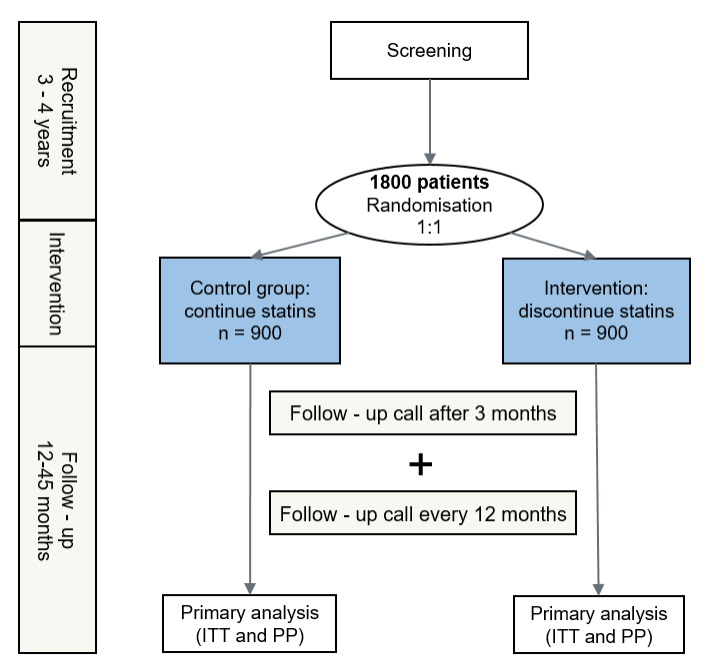
Flowchart of the STatin discontinuation as pRevention among the Elderly And Multimorbid Study. ITT, intention-to-treat; PP, per-protocol.

STatin discontinuation as pRevention among the Elderly And Multimorbid (STREAM) is an international, multicentre, randomised, non-inferiority, parallel and controlled trial. Study subjects are randomly assigned in a 1:1 ratio to either discontinue (intervention arm) or continue (control arm) statin and other lipid-lowering drug (LLD) therapies (figure 1 and table 1). This study protocol follows the Standard Protocol Items: Recommendations for Interventional Trials guidelines.^33^ The governance structure includes a steering committee and an international advisory board (online supplemental appendix 1). The sponsor, according to regulations, is Inselspital, Bern University Hospital.

**Table 1:** Study population, intervention control and outcomes

Population	Older adults (≥ 70 years) with multimorbidity (≥ 2 chronic medical problems) and statin therapy in primary prevention.
Intervention	Patients will be randomised 1:1 into the intervention or control group. In the intervention group, statin therapy will be stopped from the next scheduled intake after randomisation. Additional lipid-lowering medication lowering LDL cholesterol (ie, ezetimibe, PCSK9 inhibitors, future novel lipid-lowering medications with lowering of LDL cholesterol demonstrated in randomised trials) will also be stopped in the intervention group.
Control	No control intervention is planned. In the control group, statin therapy will be continued as prescribed before the trial.
Outcomes	The primary outcome is a composite endpoint of major CV events (non-fatal myocardial infarction, non-fatal ischaemic stroke) and all-cause death over a follow-up duration of 2 years.Secondary outcomes are: All-cause death, non-CV death, major CV events, coronary and peripheral artery revascularisation. Geriatric-relevant questionnaires: EQ-5D questionnaire, verbal numeric pain rating score, falls, SARC-F questionnaire and Girerd Medication Adherence Scale.

CV, cardiovascular; EQ-5D, European Quality of Life 5-Dimensions; LDL, low-density lipoprotein; SARC-F, Strength, Assistance with walking, Rise from a chair, Climb stairs and Falls.

### Objectives

The overall aim of this study is to assess the safety of discontinuing statin therapy compared with continuing statin therapy in older adults with multimorbidity and without clinical CVD. The primary objective is to compare a composite endpoint of major CV events and all-cause death between the control and intervention groups.

The secondary aim is to compare the impact of statin discontinuation on geriatric symptoms. Specific secondary objectives also include all-cause death (including CV and non-CV deaths) and geriatric endpoints, such as falls, health-related QOL, muscle pain and sarcopenia.

### Study setting

The trial is conducted across multiple large hospitals and general practitioner (GP) practices in Switzerland, Bordeaux (France) and Leiden (Netherlands). Detailed information regarding specific study sites can be found in the official record on clinicaltrials.gov (NCT05178420). Multiple general practices also refer patients for enrolment to investigators’ sites.

### Eligibility criteria

Inclusion criteria are patients ≥70 years of age, with multimorbidity defined as ≥2 coexistent chronic conditions (besides dyslipidaemia) with an estimated history of 6 months or more based on clinical decision^10^ and taking a statin for ≥80% of the time during the year before baseline according to the patient. We did not include an upper age limit because we aimed to include all older patients to ensure broad generalisability of our findings.

Exclusion criteria are as follows:

- Secondary prevention settings based on previous large statin trials^4 34^:
  - History of myocardial infarction type 1 (Non-ST-elevation myocardial infarction/ST-elevation myocardial infarction)
  - Stable angina pectoris with documented ischaemia on a stress test or with significant coronary disease defined as a coronary stenosis >50%.
  - History of percutaneous coronary intervention (balloon or stent) or coronary artery bypass graft.
  - History of unstable angina, defined as acute coronary syndrome symptomatic at rest, crescendo or new-onset angina (Canadian Cardiovascular Society Score 2 or 3) without ECG or cardiac biomarker changes (based on available documents).
  - History of ischaemic stroke (this does not apply to clearly cardioembolic causes for stroke).
  - History of transient ischaemic attack, defined as transient neurological deficit without diffusion restriction in MRI.
  - History of carotid revascularisation (eg, stent, bypass, carotid thrombendarterectomy)
  - History of peripheral arterial disease requiring revascularisation (eg, percutaneous transluminal angioplasty, stent, femoral thrombendarterectomy, bypass) or Fontaine IV
- Aortic disease that required a vascular repair or aortic aneurysm with a maximum diameter >5.5 cm (men) or >5.2 cm (women) based on available documents
- Probable familial hypercholesterolaemia based on Dutch lipid score ≥6 based on available documents (low-density lipoprotein cholesterol (LDL-C), family history, personal history)^35^
- Elevated risk of death within 3 months after baseline, defined as:
  - Hospitalised patients planned for palliative care within 24 hours of admission.
  - Hospitalised patients with a Palliative Performance Scale level <30%, based on the situation at least 1 month before hospitalisation, this corresponds to an estimated survival of 43% after 3 months.^36^
  - Patients with an advanced metastatic cancer prognosis of ≤20% survival rate within 1 year after baseline (based on an online tool: https://cancersurvivalrates.com).
- Participation in a clinical trial with potential impact on the STREAM CV endpoints, based on clinical judgement.

Individuals with subclinical CVD, such as coronary sclerosis or carotid plaques, are not excluded, as they were not specifically included in large secondary prevention statin trials. We do not exclude patients with diabetes, as RCTs did not find clear benefits in primary prevention in patients beyond 70 years of age.^11 37^ Additionally, patients with cognitive impairment or psychiatric diseases are not excluded to increase generalisability.^38^

### Interventions

In the intervention group, statin therapy is stopped from the next scheduled intake following informed consent and randomisation. Additionally, all lipid-lowering medications (ie, ezetimibe, PCSK9 inhibitors, bempedoic acid and future novel lipid-lowering medications with lowering of LDL-C demonstrated in randomised trials) are also stopped in the intervention group. In the control group, statin therapy and other LLDs are continued as per usual care.

### Assessment of primary outcome

The primary endpoint is a composite endpoint of adjudicated major non-fatal CV events (non-fatal myocardial infarction, non-fatal ischaemic stroke) and all-cause death over a follow-up duration of at least 2 years. All-cause death (and not CV death only) was chosen to account for competing mortality among this multimorbid population and a possible shift from CV to other causes of death (such patterns were seen in two statin RCTs).^3 8 39^ The composite endpoint was selected to assess the net clinical benefit in this population with expected high mortality.

### Assessment of secondary outcomes

The following secondary outcomes will be evaluated:

- All-cause death.
- Non-CV death.
- Major CV events (CV death, non-fatal myocardial infarction and non-fatal ischaemic stroke).
- Total CV events (CV death, non-fatal myocardial infarction, hospitalisation for unstable angina, non-fatal ischaemic stroke (including TIA) and arterial revascularisation (coronary and peripheral urgent and non-urgent revascularisation)).
- Total composite events (all-cause death, non-fatal myocardial infarction, hospitalisation for unstable angina, non-fatal ischaemic stroke (including TIA) and arterial revascularisation (coronary and peripheral urgent and non-urgent revascularisation)).
- Sarcopenia parameters, measured by Strength, Assistance with Walking, Rising from a Chair, Climbing Stairs and Falls Questionnaire at 12 months, 24 months, 36 months and 48 months.^40 41^
- Girerd medication adherence scale at 0 months, 12 months, 24 months, 36 months and 48 months.^42^
- EQ-5D Questionnaire at 3 months, 12 months, 24 months, 36 months and 48 months.^43^
- Verbal Numeric Pain Rating Score at 3 months.^44 45^
- Self-reported falls at 12 months.

As the only previous RCTs evaluating statin discontinuation in older adults showed slightly increased QOL,^18^ these geriatric questionnaires were added to evaluate the impact of statin discontinuation on geriatric syndromes. We will collect sociodemographic data, including marital status and receipt of formal or informal caregiving support. Additionally, at baseline, we will assess frailty using the FRAIL Scale^46^ and cognitive function using the Six-Item Cognitive Impairment Test Score.^47^ This will allow for secondary analyses and exploration of potential effect modifiers in our results.

### Study duration

The expected recruitment duration is about 36 months. Study participants remain in the study for the entire duration of the intervention period. Minimum intervention period duration is 12 months, and the maximum intervention period duration is 48 months. The mean intervention period is expected to be 24 months.

### Follow-up

Follow-up visits will be conducted at 3 months and 12 months after randomisation, then yearly until end of study with a maximum follow-up of 48 months after randomisation. Study personnel will contact the participant and/or GPs by phone and will review medical records to obtain information from hospitals and GPs about medical events and drug treatments during follow-up. We will collect the dosage and frequency of lipid-lowering agents. For concomitant CV treatments, the drug names will be recorded and if they have been established since baseline or started within any of the follow-up periods. At each yearly follow-up call, we will assess the Girerd Medication Adherence Scale. This approach enables continuous monitoring of intervention adherence throughout the study. Additionally, patients and their GPs will be reminded of the randomisation assignment after each follow-up call and instructed to inform the study personnel of any changes in lipid-lowering treatment. Medication adherence will be further assessed with a lipid-level measurement in 100 patients per group at the 1-year follow-up. Patients are not excluded if they are hospitalised during the follow-up period; all participants remain in the study regardless of hospitalisation. All hospitalisations are recorded within the safety reporting, and those potentially related to a CV event or death are adjudicated to ensure accurate outcome assessment.

### Sample size

The sample size calculation is based on a non-inferiority design where we test whether discontinuing statins does not result in reduced event-free survival time (primary endpoint) up to a certain degree (non-inferiority margin). The primary effect measure will be the difference in the restricted mean event-free time over 24 months between the two arms.^48 49^

We defined the non-inferiority margin on the absolute scale of integrated risk difference and fixed it at 5% over 24 months of follow-up. This can be interpreted as follows: we want to exclude a loss in event-free time of more than 2 ½ weeks per year (or about 1.5 days per month) over a 2-year observation period (5.2 weeks overall). This difference was considered clinically irrelevant (=non-inferior) given the multimorbid study population with a high event and death risk by all project partners and our international advisory board (online supplemental appendix 1).^50^

Based on preliminary data in the beginning of 2020 from our OPERAM trial that also randomised a multimorbid population over 70 years old,^38 51^ we estimated the primary event probabilities in the control group at landmark time points as follows: 22% at 12 months, 34% at 24 months and 43% at 36 months; a drop-out rate of 2% over 1 year was observed.^38^ With 1800 participants, we will achieve a power of 89% in the intention-to-treat (ITT) and 83% in the approximated per-protocol (PP) analysis set at a one-sided α level=0.025.

### Recruitment

The participating study centres screen their electronic patient records in line with the local regulations on data protection for patients fulfilling the eligibility criteria. GP practices have also been contacted and asked to screen and inform their patients about the possibility to participate in the study. Patient and public involvement in recruitment is described in the corresponding section. For screened patients who do not consent to participate, the local sites are advised to collect age, gender, eligibility criteria assessment and the reason for not consenting systematically in the electronic case report form to assess the degree of potential selection in the study enrolment process.

### Allocation

Participants are randomised in a 1:1 ratio to either discontinuing statin versus continuing statin, with stratification according to age (categorised into two groups: cut-off <75/≥75 years of age), statin intensity (categorised into high, moderate and low intensity)^25^ and time under statin before inclusion (cut-off: ≤6/>6 years, as 6 years is the longest follow-up in statin trials published in the CTT meta-analysis)^4^ using computer-generated randomly permuted blocks of varying size. Randomisation is implemented in a web-based system that also hosts the electronic case report form. Participants are only randomised after eligibility criteria have been checked and informed consent provided (online supplemental appendix 2: participant consent form). Randomisation lists were generated by an independent statistician who is otherwise not involved in the trial and only accessible by data managers who are not involved in any enrolment activities.

### Blinding

This trial is designed as an open-label study, with blinded outcome ascertainment and adjudication.

Regular telephone calls are made by partially blinded study nurses. Partial blinding means that study personnel initially assess participants’ outcome events over the phone without knowledge of their group assignment. This includes some subjective secondary outcomes, such as muscle pain. Blinding is maintained until the assessment of medication compliance, at which point group allocations become known. The primary outcome is also adjudicated by a blinded clinical event committee (CEC) (see Harms section). Blinding participants and treating physicians was deemed unfeasible for several reasons. First, providing multiple placebo tablets of identical appearance and taste for every statin agent and dosage would be impractical and costly. Second, medication changes to a standard dosing of statins in an older population might lead to increased risks of non-adherence and medication-related adverse events.^52^

Pragmatic studies often avoid placebos intentionally, aiming to assess the difference between an intervention and the best current usual treatment. Most large RCTs on deprescribing do not use placebos.^18 53^

### Data collection and management

For each enrolled trial participant, an electronic case report form is maintained, using a dedicated EDC system (https://webspirit.systems/).

### Statistical methods

The primary statistical analysis of the trial will be done at CTU Bern, based on well-established standard operating procedures. Details are specified in a statistical analysis plan (SAP) that has been finalised before all patients are enrolled.

The primary outcome of STREAM is analysed with a flexible parametric survival model with all stratification factors as covariates. The difference in restricted mean event-free time over 2 years will be the effect measure.^48 49^ A 2-year period was chosen, as this is the average follow-up time in this trial and a clinically relevant timeframe given the old multimorbid patient population with expected high mortality by expert consensus of the steering committee and our international advisory board. Two analysis sets are used for the primary analysis as described below. Non-inferiority will be declared if, for both ITT and PP analysis sets, the one-sided 97.5% CI does not cross the non-inferiority margin. We acknowledge that any choice for the non-inferiority margin is to some extent subjective, and a margin that is universally agreeable is not achievable, especially in this heterogeneous patient population.^54 55^ Components of the primary outcome and other time-to-event outcomes are analysed with the same methods as the primary outcome. Effects on time-to-event outcomes may also be presented as HRs. Continuous outcomes will be analysed using linear regression with baseline values and stratification factors as covariates and mean difference as effect measure.

ITT and PP analyses will be performed. The ITT analysis set consists of all randomised patients in the allocated group regardless of any protocol violations or crossover. For the PP analysis set, we defined criteria in the SAP for protocol adherence and postrandomisation factors that might cause confounding/selection bias.^56^ For this, we considered prerandomisation and postrandomisation factors that are predictive of adherence, amount of statin intake that constitutes adherence to each arm, crossovers, other CV active medication and intercurrent events. Patients who deviate from their assigned treatment for more than two consecutive months will be classified as crossovers. Rather than excluding these individuals from the PP analysis and introducing selection bias, their contribution to the final model will be down-weighted using inverse probability weighting. The 2-month threshold was selected because exact start and stop dates of treatment deviations cannot be reliably determined from follow-up calls. This corresponds to an adherence rate of 80%, which is considered sufficient.^57 58^ Moreover, given that the effects of statin therapy on risk are not immediate, a temporary interruption of this duration is considered tolerable.^38 59^ PP analyses will be done using g-methods adapted to estimate the restricted mean event-free time.^60^

Participants without a time-to-event outcome reported as a serious adverse event (SAE) will be assumed not to have had an event. Dropouts and loss to follow-up will be censored at the time point of the dropout. For other endpoints, multiple imputation will be considered if the proportion of missing data is between 5% and 40%.^61^ Multiple imputation will use logistic regression for binary outcomes and predictive mean matching otherwise. 50 imputation datasets will be created and results pooled using Rubin’s rule. Imputation models will use treatment group, selected baseline parameters as well as selected endpoints. Times until events occurred will not be used, but the binary indicators may be used in the models.

Subgroup analyses will be performed by statin intensity, statin duration, sex, age, frailty, history of diabetes, recruitment source, CV risk, smoking status, degree of multimorbidity and hypertension. However, given the limited accuracy of life expectancy models in older, multimorbid patients, we chose not to use life expectancy but rather stratify the analysis according to their age.^62^

The SAP will outline the approach for addressing co-interventions using sensitivity analyses.^63^

### Data monitoring

Data are monitored centrally according to a predefined monitoring plan, with queries posted for missing and/or inappropriate data. On-site monitoring is performed according to a risk-based strategy. Safety is descriptively monitored throughout the trial by an independent international data and safety monitoring board (DSMB). The DSMB members are unblinded and independent of the study team. Formal event-driven interim analyses are performed to assess intervention safety (discontinuing statins). The main safety concern is the increased risk of CV events and all-cause death in the experimental arm. Therefore, the primary endpoint is monitored. If there is statistical evidence that the proportion of primary outcome events in the experimental arm is higher than 50% based on predefined cut-off limits, the DSMB will be informed to discuss possible adaptation of inclusion criteria (eg, increase of the minimum age if there is a signal in those under 75 years of age) for safety reasons or, in the worst case, termination of the trial, also for safety reasons. For quality assurance, the sponsor, the ethical committee or an independent trial monitor may visit the research sites for an audit.

### Harms

The follow-up assessment is conducted centrally by a team in each main centre per country (Bern, Leiden, Bordeaux). The assessment of SAEs is handled by the respective central teams in each country, even for patients recruited at local sites. The central teams are responsible for performing causality assessments to determine if the SAE is potentially related to the trial intervention (eg, major CV events and all deaths). This process involves a thorough review of the event in the context of the trial’s parameters. A CEC, comprising experts from diverse specialties, for example, cardiology, neurology, internal medicine and geriatrics, is established to perform blinded outcome adjudication of clinical events identified by the central team as suspected SAEs and to determine which events meet the trial’s definition of the primary outcome. Each suspected CV event and all deaths are independently reviewed by two adjudicators who remain unaware of the participants’ group allocation. In the case of disagreement, one of the adjudicators will provide a detailed explanation for the chosen outcome, which will be reviewed by the other adjudicator. If this leads to consensus, the agreed classification will be adopted. If on discussion, the two adjudicators remain in disagreement, a third adjudicator will independently review the event blinded to the prior adjudication results. The third adjudicator’s assessment will be final. The CEC is blinded to group allocation (information on statins, other LLDs and lipid levels are removed from documents to review to maintain blinding).

### Patient and public involvement

Patients were integrally involved in several stages of developing the core outcome sets. Specifically, semistructured interviews were conducted with older patients and caregivers to identify the most relevant outcomes for this population. Public advertisement, magazines, newspapers, the trial’s website (www.statin-stream.ch) and social media will also allow potentially eligible patients to contact the study sites directly in case they seek to participate. To minimise the burden of the intervention on very old and sick patients, special adaptations were planned. These adaptations included printable versions of the questionnaires and priority lists to reduce the follow-up calls. These measures were based on feedback from the first patients enrolled. For patients with cognitive impairments, a tailored process was defined, which allowed study participation with support from their relatives. Additionally, a patient representative is included in the DSMB to provide a patient perspective on the trial’s safety. The study results will be disseminated to patients through newsletters and press releases.

### Ethics and dissemination

The local ethics committee at each site has approved the study protocol and other documentation concerning the study conduct (Ethics Committees in Switzerland (Kantonale Ethikkommission Bern 2021–01513), France (Comite de Protection des Personnes Est I, 22.04747.000158– IDRCB 2022-A02481-42- 1°HPS) and the Netherlands (Medisch-Ethische Toetsingcommissie Leiden/Den Haag/Delft NL-007060, P23.027)).

Where needed, approval by regulatory authorities has also been obtained before enrolment of the first patient. All participants and their data are handled according to the ethical principles of the Declaration of Helsinki.^64^ This study complies with all applicable standards of the International Council for Harmonisation of Technical Requirements for Pharmaceuticals for Human Use - Good Clinical Practice guideline.^65^ The ethics committees and regulatory authorities receive annual safety reports and will be informed about study stop/end in agreement with local requirements. STREAM embraces an open access policy and strives for complete dissemination of the analysis dataset, clinical results and publications.

## Discussion

As there is limited evidence concerning the benefits of statins in primary prevention among older adults, with potential negative effects such as interactions or side-effects associated with statins,^17^ and only a small RCT at end of life about discontinuing statins,^18^ the STREAM trial will provide robust evidence on these research questions.

The design of the STREAM trial has several strengths. First, in order to increase generalisability, the STREAM trial is designed as a multicentric study and involves direct recruitment of participants in GPs’ offices. Second, the STREAM trial focuses on older adults with multiple chronic conditions and will fill a gap in understanding the benefits and risks of statin therapy in this understudied population.^6^ Lastly, the STREAM trial incorporates a primary outcome composed of hard clinical endpoints—a composite of major CV events and all-cause mortality—alongside secondary patient-centred outcomes such as QOL and muscle function, which provide insights beyond CV outcomes.

While the open-label approach offers advantages such as pragmatism, logistical efficiency and direct implementation of findings into clinical practice, it also poses certain limitations. Participants might cross over into the other study group. To address this, regular monitoring and follow-up will assess if participants adhere to their assigned treatments, and we will remind the treating GP at every follow-up of the participant’s treatment allocation. Statistically, we will perform several predefined PP analyses to address this issue. Second, given the open-label design, co-interventions may occur^63^ and will be monitored and addressed, if important differences between both groups arise, as predefined in the SAP. Third, the open-label design might influence the assessment of geriatric secondary outcomes. These outcomes are therefore assessed in a partially blinded manner to lower the risk of bias. In addition, the detection of outcomes will be primarily patient-reported, which might lead to missing data. Missing data will be mitigated by contacting treating physicians for additional information if the patient is not able to answer all follow-up questions. However, the primary outcomes are composed of hard endpoints and not subject to the placebo effect, thus we expect only limited potential bias. An independ ent adjudication committee composed of clinicians, who are not involved in the inclusion or follow-up of STREAM participants and remain blinded to study group allocation, will assess and adjudicate all CV serious adverse events.

Only one trial is currently examining the discontinuation of statins for primary prevention in patients aged over 75 years.^66^ Our trial will have an extended follow-up period and incorporate more patient-centred secondary outcomes. Another ongoing trial is examining statin continuation versus placebo in older patients; however, this trial is not including multimorbid, frail patients.^67^ The STREAM trial will therefore be able to inform future guidelines on the safety and feasibility of discontinuing statins in older adults with multimorbidity. This could help reduce polypharmacy and potentially inappropriate statin prescription, which is highly prevalent in this patient population.^68^ Reducing inappropriate statin therapy will potentially improve the QOL and minimize the risk of adverse drug reactions.^69 70^

The study’s findings will contribute insights into the management of statin therapy among older adults, potentially informing clinical practice and guiding future research.

### Current status of the STREAM trial

The STREAM-trial started recruitment in November 2021 and is expected to be complete in early 2025. The trial follow-up is expected to be completed in 2026.

## Supplementary material

10.1136/bmjopen-2024-093833 online supplemental appendix 1

10.1136/bmjopen-2024-093833 online supplemental appendix 2
