# Supplementary material for: Rationale and design of ‘discontinuing statins in multimorbid older adults without cardiovascular disease (STREAM)’: study protocol of a randomised non-inferiority clinical trial
Source: BMJ Open. 2025 May 23;15(5):e093833. doi: 10.1136/bmjopen-2024-093833 (PMC12104904; doi:10.1136/bmjopen-2024-093833)
Supplement: online supplemental appendix 1 [file bmjopen-15-5-s001.docx]

# Appendix

Appendix 1: International Advisory Board:

- Prof Douglas C. Bauer, MD, Department of Medicine, Epidemiology and Biostatistics,

University of California, San Francisco, USA; role: expert in clinical trials

- Prof Fabrice Bonnet, MD PhD, Department of Internal Medicine, Saint-André Hospital, Bordeaux

University Hospital, France; role: expert in statin withdrawal trials

- Prof Philip Greenland, MD, Harry W. Dingman Professor, Dpt Preventive Medicine, Feinberg

School of Medicine, Chicago, USA; role: expert in statins and lipid guidelines

- Prof Rita Redberg, MD, Department of Cardiology, University of California, San Francisco, USA; role:

expert in cardiology and statins
