# Supplementary material for: Rationale and design of ‘discontinuing statins in multimorbid older adults without cardiovascular disease (STREAM)’: study protocol of a randomised non-inferiority clinical trial
Source: BMJ Open. 2025 May 23;15(5):e093833. doi: 10.1136/bmjopen-2024-093833 (PMC12104904; doi:10.1136/bmjopen-2024-093833)
Supplement: online supplemental appendix 2 [file bmjopen-15-5-s002.pdf]

Request to participate in medical research:

Study on the benefits of statin therapy in older people without a pre-existing heart attack or stroke

Dear Sir or Madam

We would like to ask you whether you would be willing to participate in our research project.

Your participation is voluntary. All data collected in this project is subject to strict data protection regulations. The research project is being conducted by Prof. Dr. med. Nicolas Rodondi, Chief Physician at the University Clinic for General Internal Medicine, Inselspital Bern and Director of the Bern Institute of Primary Healthcare (BIHAM).

We will explain the most important points to you and answer your questions. So that you can already get an idea, we present the most important points here in brief. This will be followed by more detailed information.

For reasons of readability, the masculine form is used when referring to persons, but the feminine form is always included.

Why are we carrying out this research project?

- Therapy with a statin is prescribed to reduce the cholesterol in the blood. In patients who have already suffered a heart attack or stroke, this can help prevent a heart attack or stroke. However, the benefit of statin therapy has not been proven in people over 70 years of age without a pre-existing heart attack or stroke. Statins can have undesirable effects. For example, muscle cramps, muscle pain or muscle weakness can occur, which can lead to falls and have a negative impact on quality of life. In addition, statin therapy increases the risk of unwanted interactions with other medications (e.g. antibiotics, medication against cardiac arrhythmia, medication to lower blood pressure, antidepressants), which can lead to possible side effects. Often side effects are not recognized as such until you try to stop taking a medication, especially if you are taking several medications. For example, muscle weakness (which can lead to falls) is thought to be due to age, although it may also be related to statin use.
- The aim of our study is to investigate the influence of statin therapy on the quality of life of people over 70 years of age.
- This study is supported by all Swiss institutes of primary healthcare as well as by general practitioners themselves. They have expressed the wish to learn more about

the benefits of statins in patients who have never suffered a heart attack or stroke in order to optimize the treatment of their patients.

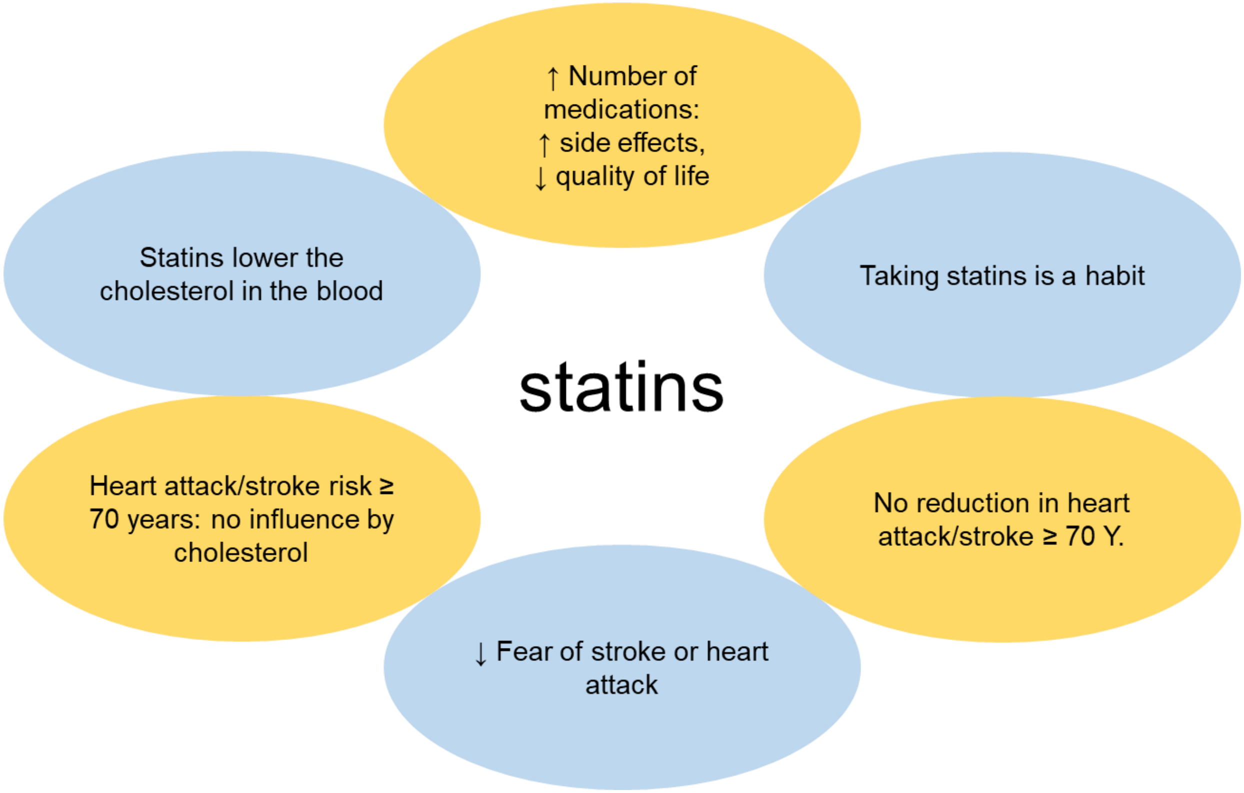

Possible advantages and disadvantages of statins in people over 70 who have not had a heart attack or stroke

What do I have to do if I take part? - What will happen to me if I take part?

- Your participation is voluntary. If you decide to take part, you will be randomly assigned to one of two groups: “Continue statin therapy” or “Stop statin therapy”.
- The study (referred to as the “intervention phase”) lasts between one and four years, depending on the time of inclusion. The first study visit usually takes place in person at one of the research centers or with a general practitioner who has received specific training in research. However, if it is easier for you, this visit can also be made by telephone. This phone call can be made by the general practitioner, a study center near you, or the central study team in Bern. After the first visit, you will only be contacted by telephone; initially after three months and then once a year until the end of the intervention phase (max. 4 years). 6 months after the end of the intervention phase, we will ask you or your general practitioner about your current cholesterol-lowering therapy and your most recent cholesterol values. After that, we would contact you annually by telephone for a follow-up check if you give us your separate consent (so-called “long-term monitoring”).

Summary of the study

Why?	Statins are often prescribed after a heart attack or stroke. Studies to date show no benefit of statins in people over 70 years of age who have not had a heart attack or stroke. Stopping statins in this case could improve quality of life. There is too little data on the benefits of statins in this rarely studied population.
Aim	Evaluation of the function of statins in people over 70 years of age who have never had a heart attack or stroke before.
Target group	≥70 years, ≥2 chronic diseases (e.g. hypertension, asthma), statin for ≥1 year, no (pre-)existing heart attack or stroke
Group Allocation	Random (by computer) division of patients into 2 groups:<br>1) Continue statin therapy<br>2) Stop statin therapy
First Visit	1 visit in person at a research center near you or at a general practitioner's office (or by phone if you wish)
Follow Up Visits	By telephone, after 3 months and then 1x/year & 6 months after completion of the intervention phase. Long-term monitoring 1x/year
Duration	1-4 years, depending on when you are included in the study

What benefits and risks are associated with this?

Benefits

- You may benefit from fewer interactions with other medications and a reduction in drug side effects if you are assigned to the “stop statin therapy” group. It is one of the aims of this study to find out if there is such a benefit. Even if you are not currently experiencing any direct side effects, it is possible that you may experience an improvement after stopping statin therapy, as side effects are not always perceived as such. For example, muscle weakness (which can lead to falls) may be thought to be age-related, whereas it may be related to taking statins.
- There is no direct personal benefit in this study.
- By participating in this study, you will help to ensure that statin therapy is used in a more targeted manner in older people in the future.

Risk and burden

- The benefit of statin therapy in older patients without existing cardiovascular disease has not been proven. One risk of stopping statin therapy is the occurrence of cardiovascular disease (myocardial infarction) if patients have recently suffered a myocardial infarction. However, this risk did not occur in patients without a heart attack or stroke.
- The progress of the study is closely monitored by a committee responsible for study safety. If significantly more cardiovascular diseases occur in one of the two groups, the study would be terminated immediately.

By signing at the end of the document, you confirm that you are participating voluntarily and that you have understood the contents of the entire document.

Detailed information

1. Aim and selection

In this research project, we are investigating the benefits of statin therapy when taken to lower blood cholesterol levels. We are investigating whether statin use can prevent heart attacks or strokes (= primary prevention) in people over the age of 70 who are multimorbid (≥ 2 chronic diseases) and have never had a heart attack or stroke. “Multimorbid” means that a person has at least two chronic diseases, such as high blood pressure or asthma. We are investigating the influence of statin therapy on quality of life, muscles, adverse drug reactions, the risk of falling and the risk of heart attack or stroke. For this purpose, we compare a group of patients who continue taking the statin with a group of patients who stop taking the statin.

We are asking you to participate in this study because you fulfill the inclusion criteria: ≥70-years-old, statin use for ≥1 year, ≥2 chronic diseases, and have never had a heart attack or stroke.

2. General information

In people who have suffered a heart attack or stroke, statin therapy reduces the risk of a second event (secondary prevention).
However, in people over 70 years of age who have never had a heart attack or stroke, the benefits of statin therapy have not been proven. However, statins can have adverse effects (e.g. muscle pain, cramps or weakness), which can lead to falls and worsen quality of life. The fact that this could be a side effect of the medication often goes unnoticed. People who are over 70 years old and have several chronic illnesses have rarely been included in studies to date. A few studies with such people have shown no benefit of statin use in relation to cardiovascular disease. In other words, neither more nor fewer diseases such as strokes or heart attacks have occurred. However, an improvement in quality of life was observed when statins were stopped.

Statins can also have adverse effects, such as muscle pain or cramps, which can lead to falls with possible trauma consequences. Renal dysfunction, liver dysfunction or cataracts are also possible side effects. These adverse effects can have a negative impact on quality of life. Often you only become aware of the adverse effects when you stop taking the medication, especially if you are taking several medications at the same time. Statins can also cause negative interactions with other medications, especially in older people who are taking several medications and suffer from several chronic illnesses.

Statins lower blood cholesterol. It is to be expected that the cholesterol level will rise after stopping the statin. However, studies have shown that cholesterol levels do not play a significant role in the risk of a heart attack or stroke in people over 70 years of age without a pre-existing heart attack or stroke. The interaction of various other risk factors (age, gender, smoking, high blood pressure) is particularly important in the event of a heart attack or stroke. One study has even shown that these other risk factors are more closely linked to heart attacks and strokes than an elevated cholesterol level in older people without a pre-existing heart attack or stroke.
If you decide to take part in the study, we will check with your general practitioner that you meet the inclusion criteria for participation in the study. You will then be randomly assigned (by a computer) to one of two groups:

1. Continuation of statin therapy (= control group)
2. Discontinuation of statin therapy and other medications that reduce cholesterol or blood lipid levels (= intervention group)

The study will be conducted at several hospitals and GP practices throughout Switzerland. The study of statin therapy (so-called “intervention phase”) lasts a total of four years, whereby we are looking for people to participate for three years. You will therefore take part in the intervention phase for between one and four years (depending on when you start the study). After that, you will

be contacted annually by telephone for a follow-up check if you give us your separate consent (so-called “long-term observation”). A total of 1,800 people will take part in the study.

We are conducting this study in accordance with Swiss law. In addition, we are observing all internationally recognized guidelines. The responsible ethics committee has reviewed and approved the study.

A description of this study can also be found on the website of the Federal Office of Public Health at www.kofam.ch (SNCTP000005172)

3. Process

If you decide to take part in the study, we will first contact your general practitioner. Once they have confirmed that you meet the criteria for participation in the study, we will organize the first appointment with you.

First appointment
If you are participating in the study, the first study visit will take place in person at a study center or with a general practitioner who has undergone special training for participation in studies. However, if it is easier for you, this visit can also be made by telephone. This telephone call can be made by your general practitioner, a study center near you, or the central study team in Bern. During this appointment, you will be randomly assigned by a computer program (“randomization”) to the “discontinuation of statin therapy” group or the “continuation of statin therapy” group. The probability of the respective group allocation is identical and is 50% in each case. We will also collect personal data (e.g. age, gender, marital status) and ask you some questions about your current state of health.
For some participants, we will take an X-ray of the heart using a computer tomogram (CT scan) and take a blood sample if the local study center is participating in this part of the study. After the intervention phase, we measure the calcium in the coronary arteries in the stored images of the CT scan. In the blood sample, we measure several blood markers (myocardial load, signs of inflammation, fatty protein). The tomography and the blood sample will provide us with additional results as to whether statin therapy can be of benefit in primary prevention. If you decide to participate in the study, you can indicate in the consent form below whether or not you agree to this additional blood test and/or X-ray examination. If you do not agree, we will refrain from performing these examination(s). However, you may still participate in the study.

Group allocation
In the “Continue statin therapy” group (= control group), your medical treatment will not change.
In the “discontinue statin therapy” group (= intervention group), the statin and other medications that lower LDL cholesterol (“bad cholesterol”) and blood lipid levels will be discontinued. This means that you stop the therapy(ies) without starting an alternative medication (e.g. placebo). To ensure that the results of the study (comparison between control and intervention group) are not falsified, it is very important that you do not take the discontinued medication again until the end of the study, which lasts 4 years. Exception: Your attending physician prescribes the cholesterol / blood lipid-lowering medication during the course of the study due to medical necessity. If you have any uncertainties or questions, you can contact us at any time (see chapter 14 “Contact person”).
We expect cholesterol and blood lipid levels to rise in patients in the “discontinuation of statin therapy” group (see “General information”). However, these values alone are not a reason to restart therapy in adults over 70 years of age without a pre-existing heart attack or stroke. We therefore ask you and your treating physicians not to check your cholesterol and blood lipid levels during the intervention phase if possible.

Involvement of medical staff and family members/acquaintances
At the beginning of the study, we will ask you to provide us with the contact details of your general practitioner and two family members/acquaintances. We will only contact family members/acquaintances if we are unable to contact or interview you by telephone several times.

We will inform your general practitioner about your participation in the study. In addition, we will ask your general practitioner to provide us with your medical data relevant to the study, which he/she collected before the study and will collect during the study, in order to complete the study data. If you obtain your medication from a pharmacy, we would also ask you for the contact details of your pharmacist so that we can also inform them about your participation in the study. If necessary, we will ask medical staff who have cared for you or will care for you during the study for your medical data relevant to the study. This may be medical staff from a hospital, a nursing home, Spitex or another medical facility. If we are unable to ascertain your state of health during the study, we may contact your municipality of residence (e.g. to obtain information about a change of residence).

Follow up

Following the first personal appointment, we will call you after three months and then once a year for the entire duration of the study (1 to 4 years) to find out about your current well-being and health. We will ask you questions about your medication intake and your quality of life. A call takes about 30 minutes. At the beginning of the call, the person calling will not know which group (with or without statin) you have been assigned to. In order not to influence the results of the study, it is important that you do not disclose this information until the end of the call. At the end of the call, the medication you are taking will be discussed and you may also talk about the group allocation (discontinuation or continuation of the cholesterol and blood lipid-lowering medication). The calls are made centrally. You will receive a call from the main study team regardless of which study center you were enrolled in. Data protection remains guaranteed.
After 12 months, we will ask the GP of some randomly selected participants from both groups to send us a blood sample to compare the cholesterol and blood lipid levels of the two groups after the intervention phase. In addition, we will ask the general practitioner of these participants for a blood sample, which we will store in a biobank (collection of biological samples and related medical data on site) for further use at a later stage. This blood sample will be used, for example, to investigate the relationship between cholesterol levels, inflammation levels and disease events. For participants who had a CT scan of the heart at baseline, a repeat CT scan may be performed after the intervention phase to examine the progression of coronary calcification since the start of the study. If you decide to participate in the study, you can indicate in the consent form below whether or not you agree to this additional blood sampling and/or CT scan. If you do not agree, we will not take the blood sample. However, you may still take part in the study.

Duration of the study

The total duration of the intervention phase is a maximum of four years. Depending on when you are included in the study, the duration of the study will vary for you, but will last at least one year. Approximately 6 months after the end of the study (end of the intervention phase), we will contact you and/or your general practitioner to ask for information about your (possible) cholesterol-lowering therapy, as well as your last measured cholesterol values.
In the consent form below, you can also indicate whether you agree to the study team contacting you or your family members/acquaintances/general practitioner/medical staff/pharmacist for a follow-up visit after the intervention phase (maximum once a year, up to a maximum of 10 years after inclusion in the study) so that we can inquire about your well-being (so-called long-term follow-up). This is important so that we can also examine the long-term effects of the study.

Discontinuation of the study

The study data is reviewed at regular intervals by an independent committee. If ethical concerns and an increased risk of heart attack or stroke in the intervention group are identified, the study would be terminated prematurely immediately. The study could also be terminated prematurely if not enough patients could be recruited to carry out the study.

Overview of Study Procedure and Data Collection

Study Procedure

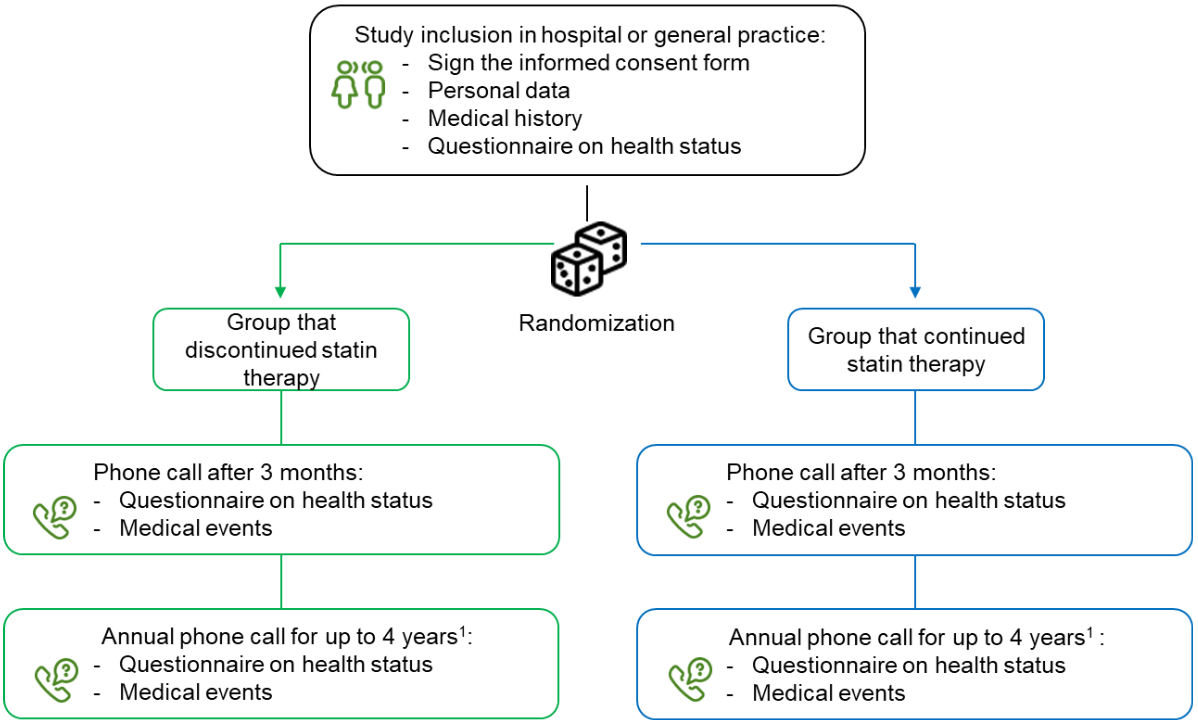

[1] Duration of the study intervention maximum 4 years; the individual duration varies from 1 to 4 years depending on the time of study inclusion. Additional annual telephone call up to 10 years after inclusion (separate consent).

4. Benefit

By participating in this study, you will help to optimize drug therapy in older people in the future by reducing the risk of interactions with other medications and adverse effects.
There is no direct personal benefit in this study. If you are assigned to the “discontinuation of statin therapy” group, you could potentially benefit from fewer

interactions with other medications, fewer drug-related adverse effects, and possibly an improvement in quality of life. Evaluating this is one of the aims of this study. Even if you are not currently experiencing any side effects, it is possible that you will notice an improvement after stopping the statin. Medication side effects are not always obvious, especially when several medications are taken at the same time. For example, muscle weakness (which can lead to falls) is thought to be age-related, although it may also be associated with statin use. If you are placed in the “Continue statin therapy” group, your current treatment will not change.

5. Voluntariness and Duties

Your participation is voluntary. If you decide not to participate in this study or later withdraw, you do not need to provide a reason. Your medical treatment/care will be guaranteed regardless of your decision.

If you participate in this study, you will be asked to:

- Adhere to the study protocol. This means that it is important that you adhere to the group assignment guidelines. In the "Continue statin therapy" group, we ask you to continue taking statin therapy as prescribed by your doctor. In the "Discontinue statin therapy" group, we ask you to stop taking statin therapy or other cholesterol- or lipid-lowering medications until the end of the intervention phase, unless these medications are re-prescribed by your

doctor during the study. You will continue taking other medications as usual, as prescribed by your general practitioner.
- Answer the questions we ask you about your study participation truthfully.
- Inform the study team in Bern (Tel. 031 632 00 69) about any medical conditions or hospital stays that occur during your study participation.

6. Risk and burden

- The benefit of statin therapy in elderly patients without existing cardiovascular disease has not been proven. One risk of stopping statin therapy is the occurrence of cardiovascular disease (heart attack) if the patient has recently suffered a heart attack. However, this risk did not occur in patients without a heart attack or stroke. In elderly people, this risk is mainly associated with other risk factors (age, gender, smoking, high blood pressure). However, such events can also occur in elderly people regardless of statin therapy. Therefore, the course of the intervention phase will be closely monitored by an independent team of experts responsible for study safety. The study data will be reviewed at regular intervals. Should the risk of cardiovascular disease be increased in a group and lead to the safety of the study no longer being guaranteed, the study would be stopped immediately.
  Your medical treatment/support is guaranteed at all times.
- An optional blood draw is associated with a very small risk of infection and bleeding.

- An optional tomography scan of the heart (CT scan) involves a slight additional radiation exposure. The unit of measurement for radiation dose is the millisievert (mSV). In everyday life, we are all exposed to natural radiation sources (e.g., radiation from rocks). On average, natural radiation exposure is approximately 3.1 mSV per year. The radiation exposure from a tomography scan is between 0.5 and 1.0 mSV per examination. This is approximately 15% of the annual radiation exposure from natural sources.

7. Questions

Participation in the study involves the risks and rewards described above. The investigator will discuss these with you during the consultation and answer any questions you may have.

8. Results

In this study there are:
1. individual study results that directly affect you;
2. individual results of the study that arise by chance (so-called random results)
3. objective final results of the entire study.

Regarding 1: During the course of the study, the investigator will inform you about all new results and findings that are personally important to you. You will be informed verbally and in writing and can then decide again whether you wish to continue participating in the study.

Regarding 2: Incidental findings are so-called "accompanying results," i.e., results that were not explicitly researched but were discovered by chance. These are results from imaging procedures (CT scans). In the case of incidental findings in CT scans, you will be informed if these findings are relevant to your health. This means that such findings will be communicated to you if a previously unknown disease is discovered by chance or if a disease that has not yet occurred can be prevented through preventive measures. If you do not wish to be informed about this (so-called right not to know), please speak to your investigator.

Regarding 3: The data we collect during the first study visit and the telephone surveys will be summarized at the end of the study to form objective final results for the entire study. Your investigator can provide you with a summary of the overall results at the end of the study.

9. Confidentiality of data and samples

9.1. Data processing and encryption

For this study, personal and health data will be collected and processed, partly in an automated format. During data collection, your data will be encrypted. Encryption means that all reference data that could identify you (name, date of birth, etc.) will be deleted and replaced with a key. Persons who do not have access to this key list cannot draw any conclusions about you. The key list always remains with the respective institution where you had the consent interview and the first study visit. Only authorized members of the research team have access to the key list. The key list is stored on a firewall- and password-protected server.

The images from the CT scans are reviewed at the respective examination site for incidental findings and then transmitted in encrypted form (just as x-rays are sent to your general practitioner or a specialist) to the central study center in Bern. Only authorized employees can view the images using their own password. Using these images, the coronary calcium score will be calculated centrally at Inselspital after the intervention phase of the study, so as not to influence the study process and results. This coronary calcium score will then be communicated to you as part of the overall study results.

We will record your contact information (address and telephone number) in a separate database, and only authorized study personnel (centralized main study team) will have access to this data in order to contact you as planned during the study.

Only a very few specialists will see your unencrypted data, and only to perform tasks related to the study. These individuals are bound by confidentiality. As a participant, you have the right to access your data.

9.2. Data protection and sample protection

All data protection regulations are strictly adhered to. It is possible that your data may need to be transmitted in encrypted form, for example, for publication, and may be made available to other researchers.

The health-related data or samples stored on-site are securely stored in a dedicated database for research purposes. Blood samples are stored in encrypted form in the Inselspital Bern biobank, in accordance with applicable guidelines and laws.

As part of this study, the doctors and pharmacists who treat you during the study may be contacted to obtain information about your health status and medications. Family members/acquaintances may also be contacted, but only if we are unable to reach you or interview you by phone several times and you have consented to this.

9.3. Data protection in the event of further use

Your data and samples may be important for answering other questions at a later date and may later be sent to and used in another database/biobank in Switzerland or abroad for yet-to-be-defined studies (further use). This other database/biobank must adhere to the same standards as the database/biobank for this study.
For this further use, we ask you to sign a second consent form at the very end of this document. This second consent form is independent of your participation in this study. You may therefore participate in the study without having to consent to the further use of your data and any samples.

9.4. Rights of access during inspections

This study may be reviewed by the relevant ethics committee or by Prof. Dr. Nicolas Rodondi, who initiated the study. The investigator will then be required to disclose your data for such review. All parties must maintain absolute confidentiality.

10. Withdrawal

You can withdraw from the study at any time. In the event of withdrawal, the data and samples collected up to that point will remain encrypted in the study documents and will be analyzed in encrypted form. This is primarily for your medical safety. Please consider whether you agree to this before participating in the study.

11. Compensation

If you participate in this study, you will not receive any compensation. Participation will not incur any costs for you or your health insurance provider. The collection and analysis of blood samples from a few randomly selected participants in the general practitioner's office will be funded by the study budget. Any follow-up costs resulting from further examinations/consultations resulting from an incidental finding will not be funded by the study budget.

12. Liability

Inselspital Bern is the sponsor of the study and responsible for its implementation. Inselspital is liable, in accordance with legal provisions, for all damages that may arise during this study. The requirements and procedures for this are regulated by law. Insel Gruppe AG has therefore taken out insurance for clinical trials and research projects with Zürich Versicherungs-Gesellschaft AG, Mythenquai 2, P.O. Box, CH-8002 Zurich.
If you suffer any damage as a result of participating in this study, please contact the investigator.

13. Funding

The study is fully funded by the Swiss National Science Foundation (SNSF).

14. Contact person(s)

You are welcome to ask questions about study participation at any time. In case of any uncertainties or emergencies that arise during or after the study, please contact:

Prof. Dr. Nicolas Rodondi
Chief Physician at the Department of General Internal Medicine, University Hospital Bern (Inselspital) & Director of the Institute of Primary Healthcare (BIHAM), University of Bern

STREAM Study Team Bern:

- Telephone number: 031 632 00 69, Monday to Friday, 8:00 a.m. – 5:00 p.m.
- Email: STREAM@insel.ch

Informed consent

Written informed consent to participate in a clinical study.

Please read this form carefully. Please ask if you don't understand anything or would like to know something. Your written consent is required to participate.

**BASEC number (after submission):**	2021-01513
**Title of the study**<br>**(scientific and layman's terms):**	Study on the benefits of statin therapy in older people without a pre-existing heart attack or stroke<br><br>*Discontinuing Statins in Multimorbid Older Adults without Cardiovascular Disease (STREAM) – a Randomized Non-Inferiority Clinical Trial*
**Responsible Institution**<br>**(Sponsor with address):**	Prof. Dr. med. Nicolas Rodondi<br>Universitätsklinik für Allgemeine Innere Medizin<br>Universitätsspital Bern (Inselspital)<br>3010 Bern
**Location:**	Inselspital Bern
**Investigator at the study site:**<br>**Last and first name in print:**	
**Participant:**<br>**First and last name:**<br>**Date of birth:**	

- I have been informed verbally and in writing by the undersigned investigator about the purpose and procedure of the study with the random allocation into one of two groups “continuation of statin therapy” or “discontinuation of statin therapy”, about possible advantages and disadvantages as well as about possible risks.
- I am participating in this study voluntarily and accept the content of the written information provided to me. I have had sufficient time to make my decision.
- My questions regarding participation in this study have been answered. I will keep the written information and receive a copy of my written consent form.
- I agree that my general practitioner will be informed about my participation in the study and will be contacted to provide medical data relevant to the study.
- I agree that other medical staff/treating physicians (for example from a hospital or nursing home)/pharmacies who cared for me before or during the study may be contacted to provide medical data relevant to the study.
- I agree that if I obtain my medication from a pharmacy, my pharmacist will also be informed about my participation in the study and contacted for information about my medication.
- I agree that my family members/acquaintances may be contacted regarding my health status if I cannot be reached by telephone.
- I agree that my local authority may be contacted if necessary (e.g. to obtain information regarding a change in living situation) if no information can be obtained from the contacts mentioned above.
- I agree that the relevant experts of the sponsor and the relevant ethics committee may access my unencrypted data for review and control purposes, but under strict confidentiality.
- I will be informed of any results and/or incidental findings in the CT scan that directly affect my health. If I do not wish this, I will inform my investigator.

- I understand that my health-related and personal data and samples can only be shared in encrypted form for research purposes for this study. The sponsor guarantees that data protection will be maintained according to Swiss standards.
- I may withdraw from the study at any time and without giving reasons. My continued medical treatment is guaranteed regardless of my participation in the study. The data and samples collected up to the time of withdrawal will still be evaluated as part of the study.
- I am aware that the Inselspital Bern's liability insurance will cover any damages.
- I am aware that the obligations stated in the information leaflet must be observed.

I consent to my general practitioner possibly (i.e., if I am randomly selected) sending a blood sample to the main study center in Bern after 12 months for cholesterol and blood lipid measurements. (Please check the box.)

☐ Yes

☐ No

I agree to participate in the long-term telephone observation after completion of the intervention phase

☐ Yes

☐ No

I agree that a blood sample will be taken upon entry into the study to determine blood markers, provided that my study center participates in this part of the study.

☐ Yes

☐ No

I agree that a CT scan of my heart may be taken upon entry into the study, provided that my study center participates in this part of the study.

☐ Ja

☐ Nein

I agree that a cross-sectional CT scan (computed tomography) of my heart may be taken after the end of the intervention phase, provided that my study center participates in this part of the study.

☐ Ja

☐ Nein

Date, Place	Signature of participant

Confirmation of the Investigator: I hereby confirm that I have explained the nature, significance, and scope of the study to this participant. I assure that I will fulfill all obligations related to this study in accordance with applicable Swiss law. Should I become aware of any aspects during the course

of the study that could influence the participant's willingness to participate, I will inform them immediately.

Daten, Place	Name and first name of the investigator<br><br><br>Signature of the investigator
