# Supplementary material for: Rationale and design of ‘discontinuing statins in multimorbid older adults without cardiovascular disease (STREAM)’: study protocol of a randomised non-inferiority clinical trial
Source: BMJ Open. 2025 May 23;15(5):e093833. doi: 10.1136/bmjopen-2024-093833 (PMC12104904; doi:10.1136/bmjopen-2024-093833)
Supplement: Supplementary file 3 [file bmjopen-2024-093833.reviewer_comments.pdf]

PEER REVIEW HISTORY

BMJ Open publishes all reviews undertaken for accepted manuscripts. Reviewers are asked to complete a checklist review form and are provided with free text boxes to elaborate on their assessment. These free text comments are reproduced below.

ARTICLE DETAILS

Title (Provisional)

Rationale and Design of "Discontinuing Statins in Multimorbid Older Adults Without Cardiovascular Disease (STREAM)" – Study Protocol of a Randomized Non-inferiority Clinical Trial

Authors

Aebi, Philipp Stefan; Adam, Luise; Haller, Moa; Bardoczi, Julia Bianca; Gencer, Baris; Bonnet, Fabrice; Beer, Jürg-Hans; Carballo, Sebastian; Christ-Crain, Mirjam; Feller, Martin; Gabutti, Luca; Haynes, Alan G; Moutzouri, Elisavet; Chocano-Bedoya, Patricia Orializ; Bassetti, Stefano; Escher, Robert; Egger, Martin; Poortvliet, Rosalinde K. E.; Schuetz, Philipp; Trelle, Sven; Wertli, Maria M.; Zekry, Dina; Méan, Marie; AUJESKY , Drahomir; Bauer, Douglas; Blum, Manuel R.; Rodondi, Nicolas

---

VERSION 1 - REVIEW

**Reviewer**	**1**
**Name**	**Gao, Yue-Xia**
**Affiliation**	**Nantong University, Department of Epidemiology and Biostatistics**
**Date**	**03-Nov-2024**
**COI**	**None**

The study protocol for the STREAM trial is well-structured and addresses an important gap in the literature regarding the use of statins in older adults with multimorbidity.

1. The study ethics code is not included in the manuscript's text.

2. Strengths and limitations of this study should contain limitations.

3. Assessment of Adherence: The protocol mentions monitoring medication adherence but could benefit from a more detailed plan on how adherence will be assessed and managed throughout the study.

4. Blinding Issues: The open-label design may introduce bias, particularly in subjective secondary outcomes. More emphasis should be placed on how the study will mitigate these biases (e.g., through partially blinded outcome assessment).

5. Handling of Missing Data: The protocol briefly mentions the potential for missing data but could elaborate on the specific statistical methods that will be employed to address this issue, ensuring robust handling of incomplete data.

**Reviewer**	**2**
**Name**	**Nielsen, Jesper Bo**
**Affiliation**	**Syddansk Universitet Det Sundhedsvidenskabelige Fakultet, Research Unit of General Practice, Dept. of Public Health**
**Date**	**06-Mar-2025**
**COI**	**None**

This appears to be a relevant and much needed study. It is a growing group of citizens in most European countries, and they are generally under-studied. The recruitment began already 4 years back and should be complete early 2025. Therefore, my comments will probably not change much, but some reflections and clarifications are needed.

1. (page 12, line 45: ‘Restricted mean event-free time over 2 years’ because ‘clinically relevant’ – Please elaborate and include some kind of reference to ‘clinically relevant’.

2. You state: ‘with expected high mortality’ – You should be able to calculate the statistically expected mortality of this age group of patients over a 2-year period? And I expect that this number will vary between the youngest (70 years) and the oldest (+ 85???).

3. Would a large difference in life-expectancy be of any importance to your study? To the way you intend to do your analyses? And do you consider age distribution when including patients in the oldest age group? (you could probably think about reporting the age distribution when reporting on the project when time comes). You may even consider including the age-adjusted estimated life-expectancy for each patient in your analyses?

4. Did you when planning consider to include an upper age limit? Why this age or why not?

5. Do you collect information on which medication (drug, dose, time period) the patients receive during the study period? If you choose not to include this information – why not?

6. Please clarify to the readers if patients are excluded if they are hospitalized during the follow-up period, and if so – all hospitalized or only those hospitalized for CVD reasons?

7. Please clarify to the readers what happens if statin for some reason is discontinued for a patient in the control arm during the follow-up time.

8. Do you include some kind of frailty score? Any reflections?

9. Do you report on physical and mental well-being besides the EQ-5?

10. Do you collect data on whether your participants live alone or with a partner as this very much affect mortality in older people? What was the reason for this choice?

11. Do you collect information whether the participants live in their own home, or whether they are institutionalized (nursing homes or otherwise taken care of by others)?

VERSION 1 - AUTHOR RESPONSE

Reviewer: 1
Dr. Yue-Xia Gao, Nantong University
Comments to the Author:

1. The study ethics code is not included in the manuscript's text.
   - Thank you for remarking this important point, we added this information on page 2 line 29 and on page 14, line 3.

2. Strengths and limitations of this study should contain limitations.
   - Thank you for pointing this out. We agree. The Strengths and limitations section was adapted to focus specifically on the methods as requested by the editor (Comment 2) and to include limitations as follows on page 5:
     - *"This study is the largest ongoing trial to assess the safety and potential benefit of statin discontinuation in multimorbid older adults, who are often excluded from major RCTs.*
     - *In addition to cardiovascular endpoints, this trial assesses patient-centred outcomes important for older adults, such as quality of life, muscle pain, sarcopenia and falls as secondary endpoints.*
     - *While outcome adjudication and ascertainment is blinded, the lack of participant and physician blinding may introduce bias in treatment adherence and subjective outcome assessments.*
     - *We engaged patients in safety monitoring, recruitment strategies and dissemination efforts, which increases the study's relevance and applicability in real-world settings."*

3. Assessment of Adherence: The protocol mentions monitoring medication adherence but could benefit from a more detailed plan on how adherence will be assessed and managed throughout the study.
   - Thank you for your comment. We have added an additional explanation on page 10, line 9-15 to clarify this aspect:
     "We will collect the dosage and frequency of lipid-lowering agents. For concomitant cardiovascular treatments, the drug names will be recorded and if they have been established since baseline or started within any of the follow-up periods. At each yearly follow-up call we will assess the Girerd medication adherence scale. This approach enables continuous monitoring of intervention adherence throughout the study. Additionally, patients and their general practitioners will be reminded of the randomization assignment after each follow-up call and instructed to inform the study personnel of any changes in lipid-lowering treatment."
   - Furthermore, we added a detailed explanation of the approach to handling cross-over due to medication non-adherence. This was added to the manuscript on page 12, line 26-33:

"Patients who deviate from their assigned treatment for more than two consecutive months will be classified as cross-overs. Rather than excluding these individuals from the per-protocol (PP) analysis and introducing selection bias*, their contribution to the final model will be down-weighted using inverse probability weighting (IPW). The two-month threshold was selected because exact start and stop dates of treatment deviations cannot be reliably determined from follow-up calls.* This corresponds to an adherence rate of 80%, which is considered sufficient.(1) *Moreover, given that the effects of statin therapy on risk are not immediate(2), a temporary interruption of this duration is considered tolerable."*

4. Blinding Issues: The open-label design may introduce bias, particularly in subjective secondary outcomes. More emphasis should be placed on how the study will mitigate these biases (e.g., through partially blinded outcome assessment).

    - We understand the needed emphasis on the mitigation of these biases. We revised the sections on page 11, line 23 - 26 for clarification and emphasis:
      " Partial blinding means that study personnel initially assess participants' outcome events over the phone without knowledge of their group assignment. This includes some subjective secondary outcomes, such as muscle pain. Blinding is maintained until the assessment of medication compliance, at which point group allocations become known."
      And page 13, line 33-35:
      "Each suspected cardiovascular event and all deaths are independently reviewed by two adjudicators who remain unaware of the participants' group allocation."
      As well as in the discussion on page 15, line 27-30.
      "An independent adjudication committee composed of clinicians, who are not involved in the inclusion or follow-up of STREAM participants and remain blinded to study group allocation, will assess and adjudicate all cardiovascular serious adverse events."

5. Handling of Missing Data: The protocol briefly mentions the potential for missing data but could elaborate on the specific statistical methods that will be employed to address this issue, ensuring robust handling of incomplete data.

    - Thank you for your comment. We added more information about how we plan to address missing data throughout the analyses on page 12-13, at line 35-5:

      "*Participants without a time-to-event outcome reported as an SAE, will be assumed not to have had an event. Drop-outs and loss to follow-up will be censored at the time point of the drop-out. For other endpoints, multiple imputation will be considered if the proportion of missing data is between 5 and 40%.(3) Multiple imputation will use logistic regression for binary outcomes and predictive mean matching otherwise. Fifty imputation datasets will be created and results pooled using Rubins rule. Imputation models will use treatment group, selected baseline parameters as well as selected endpoints. Times until*

events occurred will not be used, but the binary indicators may be used in the models."

Reviewer: 2
Prof. Jesper Bo Nielsen, Syddansk Universitet Det Sundhedsvidenskabelige Fakultet

1. Comments to the Author:(page 12, line 45: 'Restricted mean event-free time over 2 years' because 'clinically relevant' – Please elaborate and include some kind of reference to 'clinically relevant'.
    - The concept of 'clinically relevant' varies depending on the specific research question, making it difficult to provide a universally appropriate reference for this particular case. Given this, the decision to define the restricted mean event-free time over two years as relevant was made by expert consensus with our steering committee and international advisory board. This decision took into account the study length but, was mainly based on the study population, who is multimorbid and at high risk of suffering from a primary endpoint event. Based on unpublished data from our OPERAM trial, the event rate up to two years for the primary endpoint in multimorbid older adults is assumed to be more than 30%.(2)
      We included an explanation on page 12, line 11-12:
      "by expert consensus of the steering committee and our international advisory board."

2. You state: 'with expected high mortality' – You should be able to calculate the statistically expected mortality of this age group of patients over a 2-year period? And I expect that this number will vary between the youngest (70 years) and the oldest (+85?). Would a large difference in life-expectancy be of any importance to your study? To the way you intend to do your analyses? And do you consider age distribution when including patients in the oldest age group? (You could probably think about reporting the age distribution when reporting on the project when time comes). You may even consider including the age-adjusted estimated life-expectancy for each patient in your analyses?
    - Based on preliminary data from the OPERAM trial (2020)(2), which included a similar multimorbid population aged 70 and older, we estimated the primary event probabilities in the control group to be 22% at 12 months, 34% at 24 months, and 43% at 36 months, with a drop-out rate of 2% per year. This is reported in the sample size calculation on page 10, line 27-32. Given the heterogeneous life expectancy within our population, we account for age distribution through stratified randomization (<75 vs. ≥75 years) and plan subgroup analyses based on age to evaluate whether treatment effects differ across age groups. While we do not explicitly incorporate age-adjusted life expectancy into our primary analyses, it could be considered in future sensitivity analyses to explore whether expected survival modifies the impact of statin discontinuation. However previous analysis from our study group found estimating of life expectancy in multimorbid older adults to be challenging.(4) Additionally, we acknowledge the importance of reporting the age distribution of enrolled participants when presenting study results, as this will provide valuable

context for interpreting outcomes in different age cohorts. We included an explanation on page 13, line 8-9:
"However, given the limited accuracy of life expectancy models in older, multimorbid patients, we chose not to use life expectancy but rather to stratify the analysis according to their age."

3. Did you when planning consider to include an upper age limit? Why this age or why not?
    - We intentionally chose not to include an upper age limit because we aimed to include all older patients to ensure broader generalizability of our findings. While higher age is naturally associated with increased mortality, the randomization process ensures that this effect is balanced across study groups, mitigating potential biases related to age differences. We added this reflection in the manuscript on page 7, line 28-29:
    "We did not include an upper age limit because we aimed to include all older patients to ensure broader generalizability of our findings."

- Do you collect information on which medication (drug, dose, time period) the patients receive during the study period? If you choose not to include this information – why not?
    - We collect the dosage and frequency of lipid-lowering agents. For concomitant treatments, the drug names are recorded and if they have been established since baseline or started within any of the follow-up periods.
    This was added in the manuscript page 10 line 9-12:
    "We will collect the dosage and frequency of lipid-lowering agents and for concomitant cardiovascular treatments, the drug names are recorded and if they have been established since baseline or started within any of the follow-up periods."

4. Please clarify to the readers if patients are excluded if they are hospitalized during the follow-up period, and if so – all hospitalized or only those hospitalized for CVD reasons?
    - Thank you for your remark. We agree that this was not mentioned clearly in the manuscript.
    We now added a paragraph to clarify, on page 10, line 17-20:
    "Patients are not excluded if they are hospitalized during the follow-up period; all participants remain in the study regardless of hospitalization. All hospitalizations are recorded within the safety reporting, and those potentially related to a cardiovascular event or death are adjudicated to ensure accurate outcome assessment".

5. Please clarify to the readers what happens if statin for some reason is discontinued for a patient in the control arm during the follow-up time.
    - Thank you for your comment. We have added an additional explanation on page 12 line 26-33 to clarify this aspect:
    "Patients who deviate from their assigned treatment for more than two consecutive months will be classified as cross-overs. Rather than excluding these individuals from the per-protocol (PP) analysis and introducing selection bias*, their contribution to the final model will be down-weighted using inverse probability weighting (IPW). The two-month threshold was selected because*

exact start and stop dates of treatment deviations cannot be reliably determined from follow-up calls. This corresponds to an adherence rate of 80%, which is considered sufficient.(1, 5) Moreover, given that the effects of statin therapy on risk are not immediate, a temporary interruption of this duration is considered tolerable. (2, 6)"

6. Do you include some kind of frailty score? Any reflections?
   - Thank you for this important point. Frailty is a key predictor of health outcomes in older adults, particularly in multimorbid populations such as those in the STREAM trial. The FRAIL Scale is included at baseline to assess frailty status and its potential impact on study outcomes in a secondary analysis. We additionally assess SARC-F as a measure of sarcopenia, which serves as an indirect proxy for frailty, given its strong association with physical decline, mobility limitations, and adverse health outcomes in older adults.(7) Furthermore, although primarily a health-related quality of life measure, certain domains in our trial (mobility, self-care, usual activities) also reflect on components of frailty. We included a short explanation on page 9, line 30-32:
     "Additionally, at baseline we will assess frailty using the FRAIL-Scale (46) and cognitive function using the 6-CIT Score.(47) This will allow for secondary analyses and exploration of potential effect modifiers in our results."

7. Do you report on physical and mental well-being besides the EQ-5?
   - We included the 6CIT (Six-Item Cognitive Impairment Test) at baseline to assess cognitive function.(8) Physical function is evaluated using the SARC-F questionnaire, which captures sarcopenia-related impairments in strength, mobility, and falls risk, along with self-reported falls at 12. Additionally, pain intensity is measured using the Verbal Numeric Pain Rating Score (VNPRS) at baseline and 3 months, providing insight into potential musculoskeletal effects of statin discontinuation. Mental well-being is assessed through the EQ-5D anxiety/depression domain, which evaluates psychological distress. We included a short explanation on page 9, line 30-32: *"Additionally, at baseline we will assess frailty and cognitive function. This will allow for secondary analyses and exploration of potential effect modifiers in our results."*
8. Do you collect data on whether your participants live alone or with a partner as this very much affects mortality in older people? What was the reason for this choice?
   - Thank you for your comment. Yes, it is recorded if a participant lives at home or in a nursing home. Also, we record if the patient receives any paid or unpaid care at home, if by a partner, by a relative or some external person. The reason to record this is that receiving care, especially in an institution is an important predictor for mortality in multimorbid elderly and will be included in secondary analyses. An explanation was added on page 9, line 29-32:
     "We will collect sociodemographic data, including marital status and receipt of formal or informal caregiving support. This will allow for secondary analyses and exploration of potential effect modifiers in our results.
9. Do you collect information on whether the participants live in their own home, or whether they are institutionalized (nursing homes or otherwise taken care of by others)?
   - Thank you for your remark. Please refer to point 8 for the answer.

References

1. Rodriguez F, Maron DJ, Knowles JW, Virani SS, Lin S, Heidenreich PA. Association of Statin Adherence With Mortality in Patients With Atherosclerotic Cardiovascular Disease. JAMA Cardiology. 2019;4(3):206-13.
2. Blum MR, Sallevelt BTGM, Spinewine A, O'Mahony D, Moutzouri E, Feller M, et al. Optimizing Therapy to Prevent Avoidable Hospital Admissions in Multimorbid Older Adults (OPERAM): cluster randomised controlled trial. Bmj. 2021;374:n1585.
3. Jakobsen JC, Gluud C, Wetterslev J, Winkel P. When and how should multiple imputation be used for handling missing data in randomised clinical trials – a practical guide with flowcharts. BMC Medical Research Methodology. 2017;17(1):162.
4. Gastens V, Chiolero A, Feller M, Bauer DC, Rodondi N, Del Giovane C. Development and internal validation of a new life expectancy estimator for multimorbid older adults. Diagn Progn Res. 2025;9(1):5.
5. Simpson RJ, Jr., Mendys P. The effects of adherence and persistence on clinical outcomes in patients treated with statins: A systematic review. Journal of Clinical Lipidology. 2010;4(6):462-71.
6. Yourman LC, Cenzer IS, Boscardin WJ, Nguyen BT, Smith AK, Schonberg MA, et al. Evaluation of Time to Benefit of Statins for the Primary Prevention of Cardiovascular Events in Adults Aged 50 to 75 Years: A Meta-analysis. JAMA Intern Med. 2021;181(2):179-85.
7. Bahat G, Ozkok S, Kilic C, Karan MA. SARC-F Questionnaire Detects Frailty in Older Adults. J Nutr Health Aging. 2021;25(4):448-53.
8. Brooke P, Bullock R. Validation of a 6 item cognitive impairment test with a view to primary care usage. Int J Geriatr Psychiatry. 1999;14(11):936-40.
